# Inflammatory Response After Elective PCI and Subsequent Outcomes in Stable Coronary Artery Disease

**DOI:** 10.3390/jcm15176557

**Published:** 2026-08-25

**Authors:** Yalcin Dalgic, Osman Muhsin Celik, Sadiye Nur Dalgic, Furkan Gencer, Mutlu Can Kabaci, Servet Batit, Aziz Inan Celik, Sabiye Yilmaz, Metin Cagdas, Ayhan Erkol, Burak Turan

**Affiliations:** 1 Department of Cardiology, Kocaeli City Hospital, 41060 Kocaeli, Türkiye; 2 Department of Cardiology, Kapaklı State Hospital, 59510 Tekirdag, Türkiye; 3 Department of Cardiology, Biruni University Faculty of Medicine Hospital, 34010 Istanbul, Türkiye; 4 Department of Cardiology, Gebze Fatih State Hospital, 41400 Kocaeli, Türkiye

**Keywords:** elective PCI, stable coronary artery disease, high-sensitivity C-reactive protein, inflammation, white blood cell count, creatinine, prognosis, internal validation

## Abstract

**Background/Objectives:** Inflammation contributes to atherosclerotic progression and may intensify after percutaneous coronary intervention (PCI). In stable coronary artery disease (CAD), whether post-procedural inflammation is prognostically distinct from baseline inflammatory status and post-procedural renal function remains unclear. **Methods:** We studied 496 consecutive patients with stable CAD undergoing elective PCI. The primary outcome was a composite of death, myocardial infarction, stroke, or repeat revascularization. Post-PCI high-sensitivity C-reactive protein (hs-CRP) and creatinine were measured 18–24 h after PCI. Multivariable Cox models used clinically prespecified covariates, with hs-CRP log-transformed (hazard ratio [HR] per doubling) and additionally adjusted for its pre-PCI level; discrimination was assessed by ROC analysis with bootstrap internal validation. **Results:** The endpoint occurred in 43 patients (8.7%) over a median follow-up of 10 months. Post-PCI hs-CRP was independently associated with the endpoint after adjustment for pre-PCI hs-CRP, post-PCI creatinine, and albumin (HR 1.43 per doubling, 95% CI 1.05–1.96, *p* = 0.025), and after further adjustment for angiographic and procedural characteristics (HR 1.54, *p* = 0.008); pre-PCI hs-CRP was not independent (*p* = 0.083). Post-PCI creatinine was independently associated in every model (HR 1.16 per 0.1 mg/dL, *p* = 0.001) and identified a largely non-overlapping high-risk group. **Conclusions:** After elective PCI in stable CAD, post-PCI hs-CRP was independently associated with adverse outcomes and provided prognostic information beyond baseline hs-CRP and procedural characteristics in the fitted models, rather than merely reflecting baseline inflammatory status. Post-PCI renal function was a complementary, largely independent risk signal.

## 1. Introduction

Inflammation is central to the initiation, progression, and clinical destabilization of atherosclerosis [1]. Even in chronic coronary disease, residual inflammatory risk continues to influence long-term cardiovascular outcomes, as illustrated by anti-inflammatory intervention trials such as CANTOS and LoDoCo2 [2,3]. Percutaneous coronary intervention remains a cornerstone of treatment in selected patients with stable CAD, but PCI itself can provoke an acute inflammatory response through endothelial injury, plaque disruption, distal embolization, and microvascular dysfunction [4], and can also affect post-procedural renal function, with prognostic consequences of its own [5].

Previous studies have shown that baseline hs-CRP, leukocyte indices, and other inflammatory biomarkers are associated with peri-procedural myocardial injury and, later, adverse cardiovascular events after PCI [4,6,7]. More recent large real-world PCI data have also supported the prognostic relevance of hs-CRP when evaluated alongside other residual-risk biomarkers, including D-dimer and lipoprotein(a) [8]. In patients with stable angina, Gach et al. reported that an increase in hs-CRP after PCI had greater long-term prognostic value than baseline or post-procedural measurements alone [9]. Earlier elective PCI studies also showed that elevated pre-procedural hs-CRP identifies patients at increased risk for subsequent ischemic events [10], while persistent or exaggerated hs-CRP elevation after PCI has been linked to restenosis and recurrent coronary events [11,12]. The baseline hs-CRP literature has not been completely uniform: one stent-era study found no association between pre-procedural hs-CRP and angiographic restenosis or target lesion revascularization [13], whereas another cohort found that hs-CRP independently predicted in-stent restenosis at both admission and follow-up [14], and a recent systematic review and meta-analysis of 19 studies found a statistically significant but modest pooled association (standardized mean difference 0.41), with substantial heterogeneity between studies [15]. Separately, post-procedural creatinine rise is independently linked to late adverse events even when baseline serum creatinine is normal [5], and, more broadly, elevated creatinine reflects diffuse vascular disease and systemic vascular risk in stable CAD [16]. These individual associations are well established. Less well characterized, however, is whether the inflammatory state measured after elective PCI adds prognostic information beyond a patient’s baseline inflammatory burden and beyond post-procedural renal function when these are examined together in the same contemporary cohort, and whether such a signal persists after adjustment for angiographic and procedural characteristics. Addressing this integrated question—rather than any single biomarker in isolation—was the aim of the present study.

The present study was therefore designed to evaluate the relationship between inflammatory biomarkers and adverse follow-up outcomes in stable CAD patients undergoing elective PCI, with particular emphasis on post-PCI hs-CRP. We aimed to determine whether post-procedural hs-CRP elevation, as well as its magnitude of change from baseline, would remain associated with the composite endpoint after accounting for baseline inflammatory status, post-procedural renal function, and angiographic and procedural characteristics.

## 2. Materials and Methods

### 2.1. Study Design and Population

**Figure 1:**
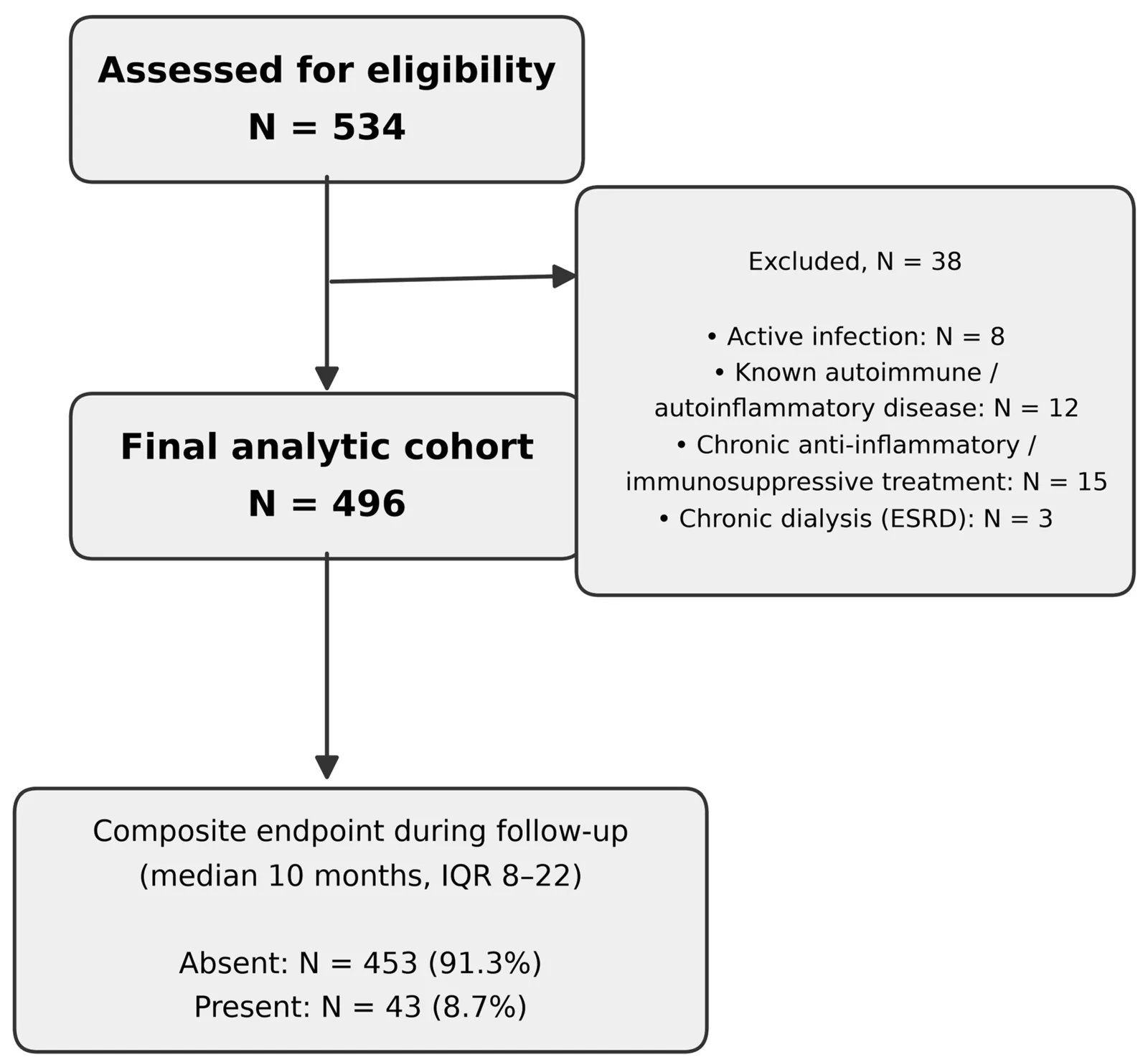
Patient enrollment flow diagram. Of 534 patients assessed for eligibility, 38 were excluded (active infection, *n* = 8; known autoimmune/autoinflammatory disease, *n* = 12; chronic anti-inflammatory or immunosuppressive treatment, *n* = 15; chronic dialysis, *n* = 3), leaving 496 patients in the final analytic cohort. The composite endpoint occurred in 43 patients (8.7%) during follow-up; the component breakdown is given in the Section 3.

This was a prospective, single-center observational cohort study. Consecutive patients older than 18 years with stable CAD undergoing elective PCI at a tertiary care center between June 2024 and April 2026 were enrolled according to a predefined protocol, with prespecified peri-procedural laboratory sampling and prospective clinical follow-up. Of 534 patients assessed for eligibility, 38 were excluded (active infection, *n* = 8; known autoimmune/autoinflammatory disease, *n* = 12; chronic anti-inflammatory or immunosuppressive treatment, *n* = 15; chronic dialysis for end-stage renal disease, *n* = 3), leaving 496 patients in the study population (Figure 1). Patients on chronic dialysis were excluded because their creatinine values do not reflect glomerular filtration and would distort the analysis of post-procedural renal function. Only elective procedures performed for stable coronary presentations were included; patients with acute coronary syndrome were not part of the population assessed for this registry. The study protocol was approved by the Kocaeli City Hospital Ethics Committee (protocol no. 2024-61) and was conducted in accordance with the Declaration of Helsinki.

### 2.2. Data Collection and Definitions

Demographic features, cardiovascular risk factors, laboratory variables, angiographic findings, procedural characteristics, and follow-up outcomes were extracted from a prospectively maintained institutional database. The primary outcome was the prespecified composite endpoint of death, myocardial infarction, stroke, or repeat revascularization during follow-up; a descriptive breakdown of how many patients met each component is reported separately in the Section 3 rather than alongside baseline characteristics to avoid comparing the composite endpoint against variables that are themselves part of its definition. The myocardial infarction component comprised patients who developed an acute myocardial infarction during follow-up, diagnosed according to standard clinical criteria consistent with the Fourth Universal Definition of Myocardial Infarction [17] and confirmed at repeat coronary angiography performed for the acute coronary syndrome (ACS) presentation. These events were not subclassified into ST-elevation (STEMI) and non-ST-elevation (NSTEMI) myocardial infarction and were identified in the database through the ACS-indicated repeat-angiography pathway rather than a separate stand-alone myocardial-infarction field. Patients who underwent repeat angiography for a suspected ACS-type presentation but in whom no angiographically significant culprit lesion was found were not counted toward the endpoint (see Section 3 and Section 4). All other individual follow-up event fields (repeat angiography for a stable-angina-type indication, death, target vessel revascularization [TVR], target vessel lesion [TVL] on repeat angiography defined as angiographically significant stenosis [>50%] in the target vessel, and intervention in other vessels) were recorded as documented in the database. Planned/staged PCI procedures were not counted toward the repeat-revascularization component: each patient was included once, and a pre-planned staged procedure was not recorded as a clinical event.

Laboratory variables included baseline hemoglobin, WBC count, platelet count, creatinine, albumin, lipid parameters, and hs-CRP, as well as post-PCI hemoglobin, WBC count, platelet count, creatinine, hs-CRP, and troponin. Post-PCI hs-CRP and post-PCI creatinine were drawn 18 to 24 h after the procedure in all patients. hs-CRP and high-sensitivity cardiac troponin were measured on a Roche Cobas 8000 analyzer (Roche Diagnostics, Mannheim, Germany). Procedural characteristics were expanded for this analysis to include vascular access route (radial vs. femoral), total fluoroscopy time, total radiation dose, and fluoroscopy frame count, in addition to number of stents, total stent length, maximum stent diameter, bifurcation PCI, and CTO PCI. All implanted stents were second-generation drug-eluting stents. Baseline (pre-procedural) use of statin, aspirin, and P2Y12-inhibitor therapy was also recorded. Calcified-lesion status and intravascular ultrasound (IVUS) use were not systematically captured in the institutional database and could not be included. Patient-level discharge medication use and adherence during follow-up were not systematically captured; institutional practice was to prescribe dual antiplatelet therapy and high-intensity statin therapy after stent implantation.

### 2.3. Statistical Analysis

Continuous variables are presented as mean ± standard deviation or median (interquartile range), as appropriate, and were compared using Welch’s *t*-test or the Mann–Whitney U test, respectively. Categorical variables are presented as counts and percentages and were compared using the chi-square test with Yates continuity correction, or Fisher’s exact test when an expected cell count was below 5. Baseline and procedural characteristics were compared according to composite endpoint status (Table 1), and a separate analysis compared patients with low versus high post-PCI hs-CRP using a 1.90 mg/L cut-off, the Youden-optimal value from ROC analysis of post-PCI hs-CRP in this cohort (Table 2). Components of the composite endpoint are reported descriptively in the Section 3 rather than as rows in the baseline comparison since comparing these against composite-endpoint status would be circular.

Event-free survival was assessed by Kaplan–Meier analysis and compared with the log-rank test, with number-at-risk tables reported beneath each curve. Cox proportional-hazards regression was used to identify independent predictors of the composite endpoint, with pre-procedural and post-procedural models evaluated separately. In contrast to the prior version of this analysis, multivariable covariates were not selected by a backward Wald stepwise procedure; instead, each model used a single forced-entry block of covariates prespecified on clinical grounds, consistent with the current recommendations that stepwise selection based on statistical significance be avoided in favor of clinically motivated variable selection [18]. The pre-PCI model included age, sex, pre-PCI creatinine, albumin, and pre-PCI WBC (*n* = 496). The post-PCI model included age, sex, post-PCI creatinine, albumin, pre-PCI hs-CRP, and post-PCI hs-CRP (*n* = 496); pre-PCI hs-CRP was added specifically to test whether post-PCI hs-CRP remains associated with outcome independently of baseline inflammatory burden, rather than simply reflecting it. A procedural-augmented model added the number of stents, total stent length, bifurcation PCI, and CTO PCI to the post-PCI model (*n* = 496). Finally, a prespecified sensitivity analysis additionally adjusted the post-PCI model for baseline statin, aspirin, and P2Y12-inhibitor use (Supplementary Table S3).

High-sensitivity C-reactive protein is strongly right-skewed and was therefore analyzed on the log scale, its conventional analytic form for this biomarker in cardiovascular research [19,20,21,22]; hazard ratios are reported per doubling (log_2_). Creatinine was analyzed on its natural scale, with hazard ratios reported per 0.1 mg/dL because its interquartile range in this cohort is far narrower than 1.0 mg/dL; after exclusion of the three chronic-dialysis patients, creatinine contained no dialysis-range extreme values and behaved as a well-conditioned linear predictor. Linearity (for creatinine) and log-linearity (for hs-CRP) were confirmed by the non-significance of an added quadratic term (all *p* > 0.20). The proportional-hazards assumption was assessed for every multivariable model using scaled Schoenfeld residuals (Grambsch–Therneau approach), with both per-covariate and global chi-square statistics reported (Supplementary Table S1) [23]. The influence of individual observations was examined with standardized dfbeta statistics from leave-one-out refitting. Because the number of events was modest relative to the number of covariates, model optimism was additionally quantified using the heuristic shrinkage factor and bootstrap optimism-correction of Harrell’s concordance index. All statistical analyses were performed using IBM SPSS Statistics, version 26.0 (IBM Corp., Armonk, NY, USA).

Exploratory ROC analysis was performed as a secondary descriptive assessment of biomarker discrimination, recognizing that it does not account for censoring or differential follow-up. Because the same sample was used to derive and evaluate each cut-off, internal validation was performed by bootstrap resampling (2000 resamples with replacement); percentile-based 95% confidence intervals are reported for the area under the curve (AUC) as a measure of internal precision and stability, which is not equivalent to external validation in an independent cohort [19]. Full bootstrap results for all four biomarkers are provided in Supplementary Table S2. A 2-sided *p* value <0.05 was considered statistically significant.

## 3. Results

### 3.1. Study Population

Of 534 patients assessed for eligibility, 38 were excluded on the prespecified grounds described in the Section 2, leaving 496 patients in the analytic cohort (Figure 1). The composite endpoint occurred in 43 patients (8.7%), whereas 453 remained free of the endpoint. Median follow-up was 10 months (interquartile range 8–22 months) overall, 10 months (8–21) in patients without the endpoint, and 16 months (7–26) in patients with the endpoint. Vascular access was radial in 392 patients (79.0%) and femoral in 104 patients (21.0%). Post-PCI hs-CRP, pre-PCI hs-CRP, and creatinine values were complete for all 496 patients, so all multivariable models are reported for the full analytic cohort (*n* = 496).

### 3.2. Components of the Composite Endpoint

Among the 43 patients meeting the composite endpoint, 11 (25.6%) died, 16 (37.2%) had a myocardial infarction confirmed at repeat angiography (not subtyped as STEMI/NSTEMI; see Section 2), 14 (32.6%) underwent repeat revascularization for a stable-angina-type indication, 10 (23.3%) had TVR, seven (16.3%) had a target vessel lesion on repeat angiography, 23 (53.5%) underwent intervention on a non-target vessel, and six (14.0%) had a stroke. Of note, a total of twenty-two patients underwent repeat angiography for a suspected ACS-type presentation, but six of these had no angiographically significant culprit lesion and were therefore not counted toward the endpoint; the sixteen counted as infarctions are those with a confirmed culprit lesion. These categories are not mutually exclusive; most patients who met the composite endpoint satisfied more than one component.

### 3.3. Baseline and Procedural Characteristics According to Composite Endpoint

Compared with patients without the composite endpoint, those with events had a higher prevalence of hyperlipidemia (79.1% vs. 58.1%, *p* = 0.012), higher pre-PCI WBC count (8.78 ± 2.74 vs. 7.76 ± 2.02 × 10^9^/L, *p* = 0.022), higher pre-PCI platelet count (265 (219–309) vs. 241 (193–278) × 10^9^/L, *p* = 0.040), higher baseline creatinine (0.94 (0.85–1.17) vs. 0.89 (0.75–1.03) mg/dL, *p* = 0.017), and lower albumin (40.3 ± 4.0 vs. 41.5 ± 3.0 g/L, *p* = 0.050). LAD PCI was numerically less frequent in the endpoint group (34.9% vs. 49.4%, *p* = 0.096). Among post-procedural variables, patients with the composite endpoint had higher post-PCI platelet counts (*p* = 0.008), higher post-PCI creatinine (0.92 (0.83–1.10) vs. 0.85 (0.73–1.01) mg/dL, *p* = 0.010), substantially higher post-PCI hs-CRP (4.3 (2.1–9.8) vs. 2.5 (1.2–5.3) mg/L, *p* = 0.001), higher Δhs-CRP (0.7 (−0.1 to 2.4) vs. 0.0 (−0.3 to 0.5) mg/L, *p* = 0.002), and higher post-PCI troponin (*p* = 0.026). Access route, fluoroscopy time, radiation dose, fluoroscopy frame count, number of stents, total stent length, maximum stent diameter, bifurcation PCI, and CTO PCI did not differ significantly (all *p* > 0.05, Table 1).

### 3.4. Characteristics According to Post-PCI hs-CRP Category

**Figure 2:**
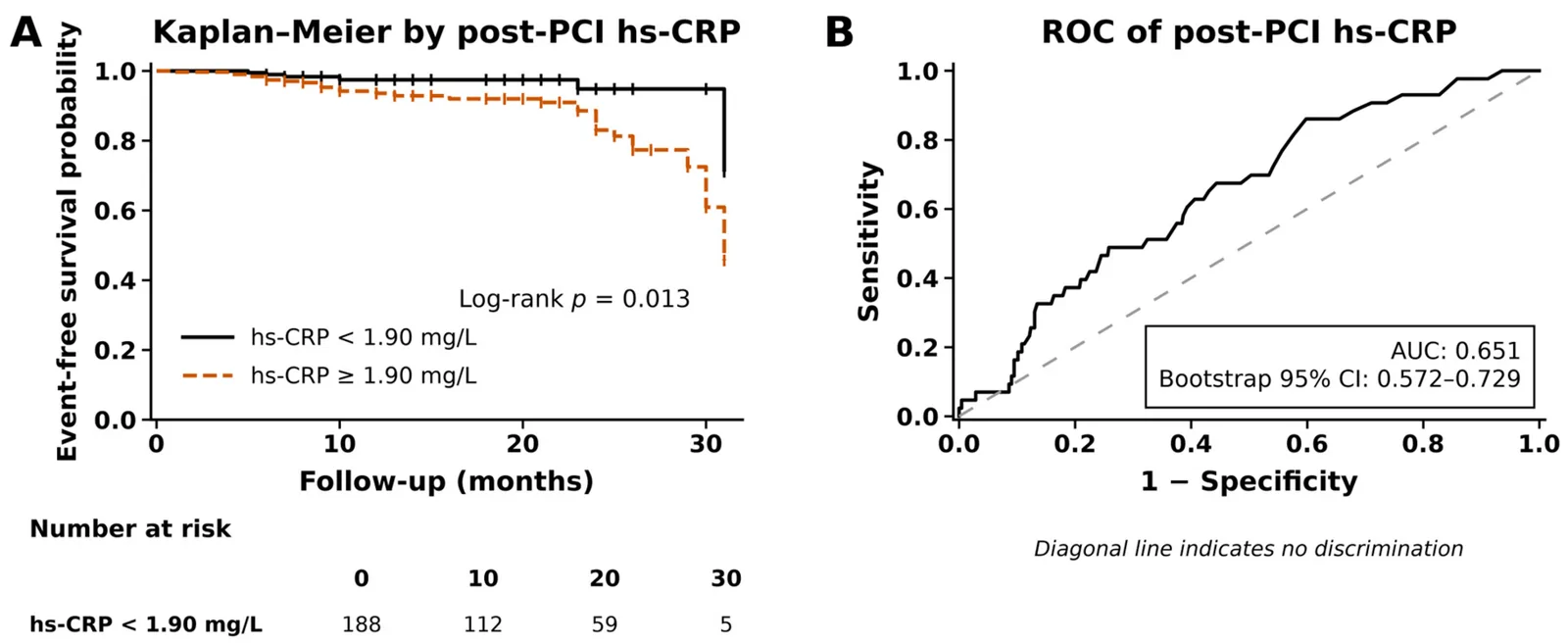
(**A**) Kaplan–Meier curves for the composite endpoint according to post-PCI hs-CRP (<1.90 vs. ≥1.90 mg/L); log-rank *p* = 0.013, numbers at risk shown beneath the plot. (**B**) ROC curve of post-PCI hs-CRP (AUC 0.651, bootstrap-validated 95% CI 0.572–0.729 from 2000 resamples); the Youden-optimal cut-off of 1.90 mg/L (sensitivity 86.0%, specificity 40.2%) was applied to the categorical analyses in panel (**A**) and the subgroup comparisons. The dashed diagonal line in panel (**B**) indicates the line of no discrimination.

When the cohort was stratified by post-PCI hs-CRP using the ROC-derived, Youden-optimal 1.90 mg/L cut-off (188 patients below, 308 at or above), patients with higher post-PCI hs-CRP were more frequently women (33.1% vs. 23.9%, *p* = 0.038) and had higher baseline inflammatory burden, including higher pre-PCI WBC count (8.16 ± 2.29 vs. 7.34 ± 1.66 × 10^9^/L, *p* < 0.001), higher pre-PCI platelet count (*p* < 0.001), higher pre-PCI hs-CRP (4.2 (2.7–8.4) vs. 1.0 (0.6–1.4) mg/L, *p* < 0.001), and lower albumin (*p* = 0.006). Importantly, pre- and post-PCI creatinine did not differ between hs-CRP groups (*p* = 0.894 and *p* = 0.653), indicating that post-PCI hs-CRP elevation and post-PCI creatinine elevation identify substantially non-overlapping patients (Table 2). The cumulative incidence of the composite endpoint was higher in the high post-PCI hs-CRP group (12.0% vs. 3.2%, *p* < 0.001), and Kaplan–Meier analysis demonstrated worse event-free survival with elevated post-PCI hs-CRP (log-rank *p* = 0.013, Figure 2A).

### 3.5. Δhs-CRP Analysis

**Figure 3:**
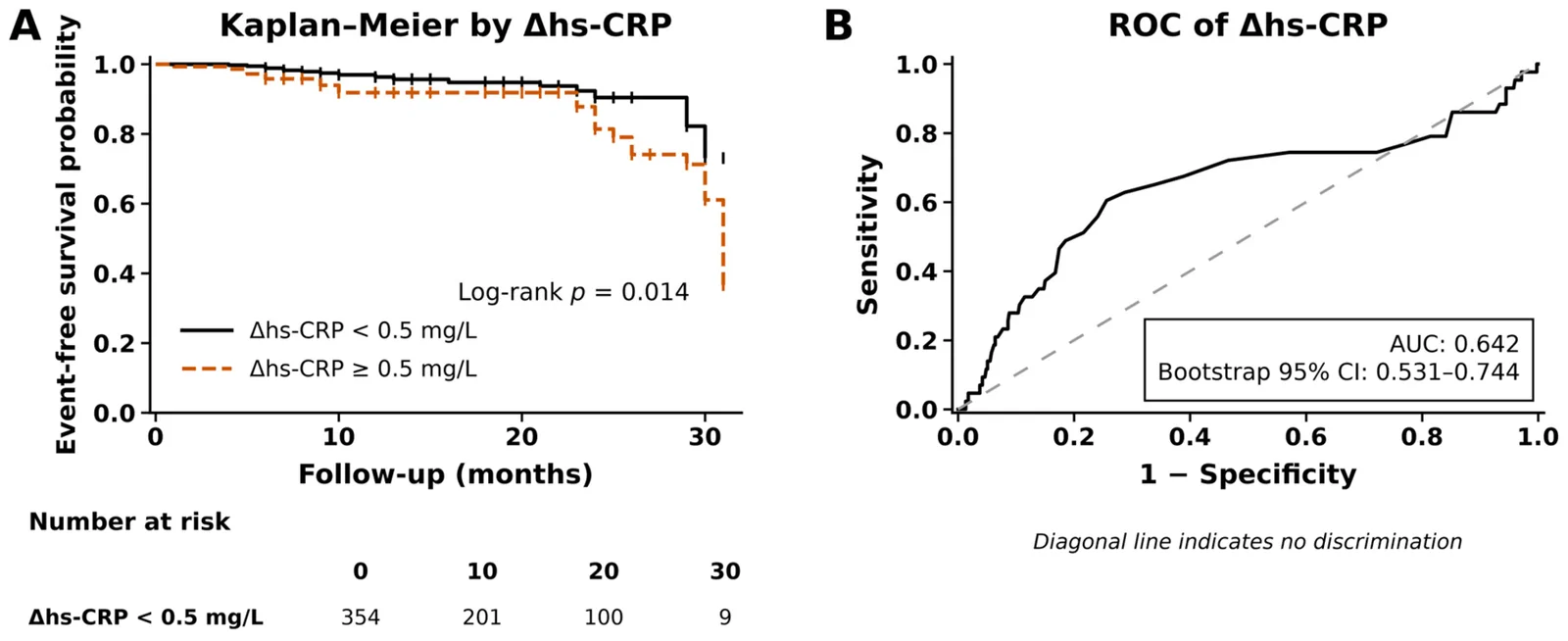
(**A**) Kaplan–Meier curves for the composite endpoint according to Δhs-CRP, post-PCI minus pre-PCI (<0.5 vs. ≥0.5 mg/L); log-rank *p* = 0.014, numbers at risk shown beneath the plot. (**B**) ROC curve of Δhs-CRP (AUC 0.642, bootstrap-validated 95% CI 0.531–0.744 from 2000 resamples); Youden-optimal cut-off 0.50 mg/L (sensitivity 60.5%, specificity 74.4%). The dashed diagonal line in panel (**B**) indicates the line of no discrimination.

The change in hs-CRP from before to after PCI (Δhs-CRP = post-PCI minus pre-PCI) was calculable for all 496 patients and was significantly higher in patients who later developed the composite endpoint (0.7 (−0.1 to 2.4) vs. 0.0 (−0.3 to 0.5) mg/L, *p* = 0.002; Table 1). Using a data-derived (Youden-optimal) cut-off of 0.5 mg/L, Kaplan–Meier analysis showed significantly worse event-free survival above this threshold (log-rank *p* = 0.014, Figure 3A), and exploratory ROC analysis yielded an AUC of 0.642 (bootstrap 95% CI 0.531–0.744, Figure 3B), numerically similar to post-PCI hs-CRP alone and higher than pre-PCI hs-CRP alone (AUC 0.563, 95% CI 0.475–0.659).

### 3.6. Exploratory ROC and Survival Analyses (Bootstrap Internal Validation)

**Figure 4:**
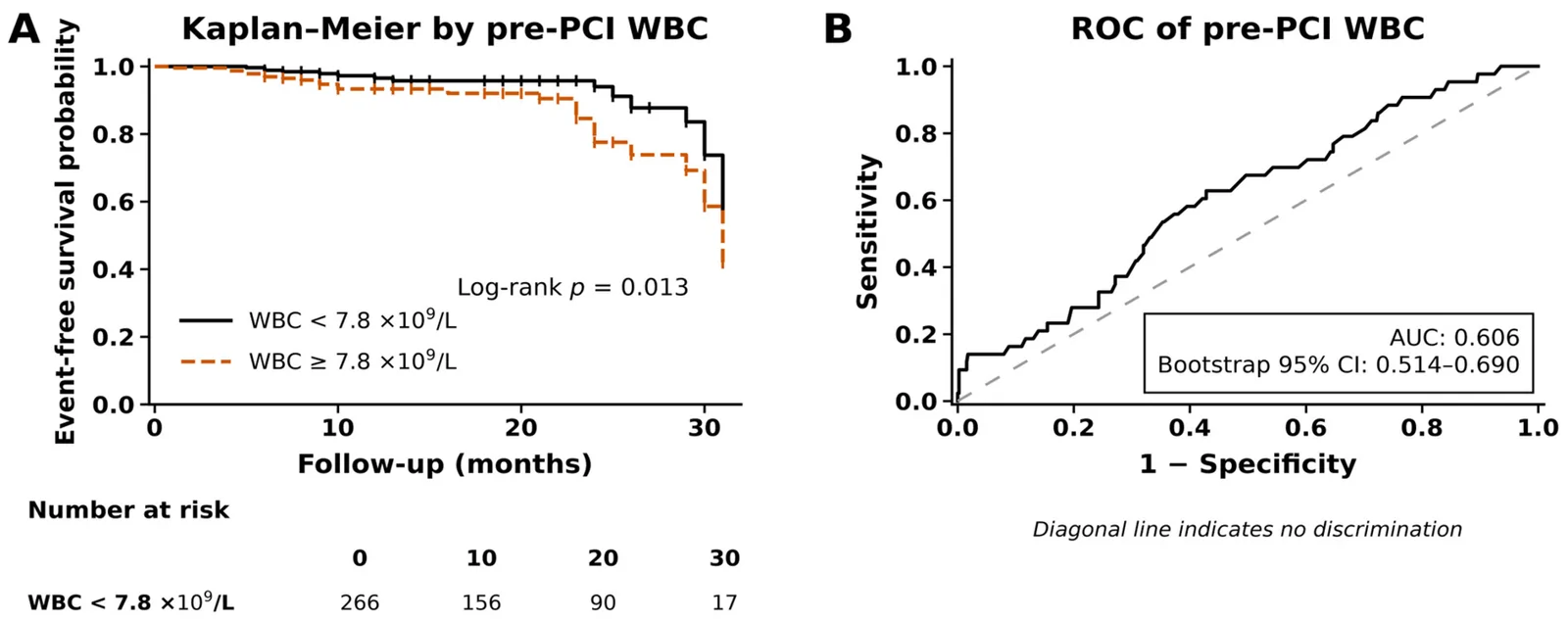
(**A**) Kaplan–Meier curves for the composite endpoint according to pre-PCI WBC (<7.8 vs. ≥7.8 × 10^9^/L); log-rank *p* = 0.013, numbers at risk shown beneath the plot. (**B**) ROC curve of pre-PCI WBC for the composite endpoint (AUC 0.606, bootstrap-validated 95% CI 0.514–0.690 from 2000 resamples); Youden-optimal cut-off 7.83 × 10^9^/L (sensitivity 62.8%, specificity 57.2%). The dashed diagonal line in panel (**B**) indicates the line of no discrimination.

Exploratory ROC analysis for pre-PCI WBC yielded an AUC of 0.606 (bootstrap 95% CI 0.514–0.690), with a Youden-optimal cut-off of 7.83 × 10^9^/L (sensitivity 62.8%, specificity 57.2%), essentially identical to the rounded 7.8 × 10^9^/L threshold used for the Kaplan–Meier analysis. Kaplan–Meier analysis using this cut-off showed poorer event-free survival above the threshold (log-rank *p* = 0.013, Figure 4A). Exploratory ROC analysis for post-PCI hs-CRP yielded an AUC of 0.651 (bootstrap 95% CI 0.572–0.729), with a Youden-optimal cut-off of 1.90 mg/L (sensitivity 86.0%, specificity 40.2%), which was the threshold used for the Kaplan–Meier and subgroup analyses. The small optimism between apparent and bootstrap-corrected AUCs for both markers (Supplementary Table S2) suggests that the apparent AUCs are reasonably stable within this sample, although this remains an internal, same-sample form of validation. Creatinine was not evaluated by ROC because it was analyzed as a continuous Cox covariate rather than via a binary threshold.

### 3.7. Multivariable Cox Regression

In the forced-entry pre-PCI model (age, sex, pre-PCI creatinine, albumin, pre-PCI WBC; Table 3, *n* = 496), higher pre-PCI creatinine (HR 1.15 per 0.1 mg/dL, 95% CI 1.06–1.25, *p* = 0.001), higher pre-PCI WBC (HR 1.15 per ×10^9^/L, 95% CI 1.02–1.30, *p* = 0.025), and lower albumin (HR 0.91 per g/L, 95% CI 0.84–1.00, *p* = 0.040) were independently associated with the composite endpoint; age and sex were not.

In the forced-entry post-PCI model that additionally included pre-PCI hs-CRP (age, sex, post-PCI creatinine, albumin, pre-PCI hs-CRP, post-PCI hs-CRP; Table 4, *n* = 496), post-PCI hs-CRP remained independently associated with the composite endpoint (HR 1.43 per doubling, 95% CI 1.05–1.96, *p* = 0.025). This directly addresses whether post-PCI hs-CRP carries prognostic information beyond baseline inflammatory status rather than simply reflecting it: once post-PCI hs-CRP was in the model, pre-PCI hs-CRP was not independently associated with outcome (HR 0.76 per doubling, 95% CI 0.56–1.04, *p* = 0.083). Post-PCI creatinine (HR 1.16 per 0.1 mg/dL, 95% CI 1.06–1.26, *p* = 0.001) and albumin (HR 0.88 per g/L, 95% CI 0.81–0.97, *p* = 0.006) were also independently associated with outcome, indicating that post-PCI hs-CRP and post-PCI creatinine each carry independent prognostic information.

Adding procedural characteristics (number of stents, total stent length, bifurcation PCI, CTO PCI) to the post-PCI model (Table 5, *n* = 496) did not attenuate either signal: post-PCI hs-CRP HR was 1.54 (95% CI 1.12–2.12, *p* = 0.008) and post-PCI creatinine HR was 1.17 per 0.1 mg/dL (95% CI 1.07–1.29, *p* = 0.001), while none of the four procedural covariates was independently associated with the composite endpoint (all *p* > 0.20). This argues against angiographic or procedural characteristics as the explanation for either signal.

Baseline statin, aspirin, and P2Y12-inhibitor use did not differ significantly between patients with and patients without the composite endpoint (Table 1). In a prespecified sensitivity analysis additionally adjusting the post-PCI model for these three medications (Supplementary Table S3), post-PCI hs-CRP (HR 1.47 per doubling, 95% CI 1.09–1.99, *p* = 0.012) and post-PCI creatinine (HR 1.15 per 0.1 mg/dL, 95% CI 1.05–1.26, *p* = 0.004) remained independently associated with the endpoint, whereas none of the three medications were independently associated (all *p* > 0.13). The primary associations are therefore not explained by baseline secondary-prevention medication use.

### 3.8. Proportional Hazards Assumption

Schoenfeld residual testing (Supplementary Table S1) showed no significant departure from the proportional-hazards assumption for any covariate in either model (all *p* > 0.05), and the global tests were also non-significant (pre-PCI χ^2^ = 7.6, *p* = 0.180; post-PCI χ^2^ = 8.9, *p* = 0.180). The linearity of creatinine and log-linearity of hs-CRP were supported (quadratic-term *p* > 0.20). hs-CRP had violated the proportional-hazards assumption on its untransformed scale, consistent with an artifact of its right-skewed distribution that resolves on the log scale; creatinine satisfied the assumption on its natural scale. Influence diagnostics showed that no observation exceeded the conventional standardized-dfbeta threshold of 1.0 (maximum 0.60), and the direction of every association was preserved on leave-one-out refitting. Given the modest number of events relative to covariates (events-per-variable 7.2–8.6), the heuristic shrinkage factor was 0.71–0.75 and bootstrap optimism in the concordance index was small (apparent c-index 0.686, optimism-corrected 0.649), indicating limited overfitting, although the coefficients should be read as estimates that may be modestly optimistic.

## 4. Discussion

In patients with stable coronary artery disease undergoing elective PCI, post-PCI hs-CRP provided a post-procedural prognostic signal that persisted after two adjustments that a purely descriptive analysis cannot address: accounting for baseline (pre-PCI) hs-CRP and accounting for angiographic and procedural characteristics. Neither adjustment attenuated the association. This is the central question raised by the finding in Table 2 that patients with high post-PCI hs-CRP already had higher baseline hs-CRP: does post-PCI hs-CRP carry prognostic information beyond the baseline level, or does it merely reflect a high baseline? The multivariable models answer this directly. When both pre- and post-PCI hs-CRP were entered together, only post-PCI hs-CRP remained independently associated with outcome (HR 1.43 per doubling, *p* = 0.025), whereas pre-PCI hs-CRP did not (*p* = 0.083). Consistently, the absolute rise in hs-CRP from baseline (Δhs-CRP) was greater in patients who developed events and separated event-free survival upon Kaplan–Meier analysis (Figure 3). Together these indicate that the post-procedural component of the inflammatory response, not simply the baseline level, carries independent prognostic weight.

Post-PCI creatinine was independently and consistently associated with outcome in every model as well, identifying a group that only partially overlaps with the high post-PCI hs-CRP group (the two markers were not significantly associated with each other in Table 2), suggesting that post-procedural inflammation and post-procedural renal function are complementary rather than redundant risk signals. This pattern is biologically plausible, though the precise mechanism cannot be established from this dataset. PCI is not only a revascularization procedure but also a controlled vascular injury that can amplify inflammatory signaling through endothelial disruption, plaque manipulation, distal embolization, oxidative stress, platelet activation, and microvascular dysfunction [4]. A higher post-PCI hs-CRP level may therefore integrate both pre-existing residual inflammatory risk and the inflammatory consequences of the procedure itself. Post-procedural creatinine elevation is, in parallel, independently associated with late adverse events even when baseline renal function is normal [5]. We deliberately avoid attributing this to contrast-induced nephropathy specifically: the classic definition of contrast-associated acute kidney injury requires a creatinine rise documented 48 to 72 h after contrast exposure, whereas post-PCI creatinine here was drawn at 18 to 24 h. We therefore describe it as a post-procedural renal-function signal, which may reflect early contrast-mediated renal stress, hemodynamic or procedural factors, or an underlying systemic vascular risk phenotype already partly captured by baseline creatinine [16]. Because patients on chronic dialysis were excluded, this renal-function signal reflects patients with native kidney function rather than end-stage renal disease, and creatinine here behaves as a graded marker of glomerular filtration rather than being dominated by a few dialysis-range extremes. This interpretation aligns with the broader concept that inflammation remains clinically relevant even in stable coronary disease, as supported by CANTOS, LoDoCo2, and COLCOT [2,3,24]. This concept has since been reinforced in contemporary statin-treated populations: in a collaborative analysis of 31,245 patients from the PROMINENT, REDUCE-IT, and STRENGTH trials, residual inflammatory risk assessed by hs-CRP more strongly predicted major adverse cardiovascular events and mortality than residual cholesterol risk assessed by LDL cholesterol [25].

An interpretive point concerns the myocardial-infarction component of the endpoint. These events were acute myocardial infarctions diagnosed on standard clinical grounds and confirmed at repeat coronary angiography; importantly, of twenty-two patients who underwent repeat angiography for a suspected ACS-type presentation, six had no angiographically significant culprit lesion and were conservatively excluded, so the infarction component reflects only patients with an angiographically confirmed culprit lesion. Because the number of patients with an acute coronary syndrome during follow-up was small (*n* = 16), we deliberately did not subclassify these infarctions into STEMI and NSTEMI or perform a subgroup analysis by infarction type, as such small strata would not support reliable inference.

Our findings are directionally consistent with the prior literature on post-procedural inflammatory activation. Gach et al. reported that in 89 patients with stable angina followed for a mean of 6.6 years, a peri-procedural rise in hs-CRP of ≥3 mg/L independently predicted major adverse cardiac events and was more predictive than pre- or post-PCI hs-CRP considered separately [9]. In the present cohort, the peri-procedural rise was likewise associated with outcome on univariable analysis (Figure 3), but after multivariable adjustment, it was the post-PCI hs-CRP level rather than the change that remained independently associated with the endpoint; this difference may reflect our substantially shorter follow-up (median 10 months vs. 6.6 years), fewer events, and a much lower Δ threshold (0.5 vs. ≥3 mg/L). Tucker et al. summarized the mechanistic and prognostic evidence linking inflammatory activation during PCI with later ischemic outcomes [4], and Zhao et al. showed that inflammatory biomarker elevation around elective PCI is associated with peri-procedural myocardial injury [6]. Earlier studies by Hoshida et al. and Saleh and Tornvall linked persistent or exaggerated CRP responses after PCI with restenosis and recurrent coronary events [11,12]. Post-PCI troponin was also numerically higher in patients who met the composite endpoint (Table 1), consistent with the dose-response literature linking peri-procedural myocardial biomarker elevation to worse outcomes [26]. More recently, Nath et al. reported that elevated hs-CRP was associated with higher six-month event rates in chronic stable angina patients undergoing drug-eluting stent PCI [27].

The pre-procedural findings also deserve attention. Higher baseline WBC count and higher baseline creatinine were independently associated with adverse outcomes in the pre-PCI model. WBC is an inexpensive, widely available marker of systemic inflammatory tone associated with later major adverse cardiovascular events after PCI [7]. Baseline creatinine likely represents a broader high-risk systemic profile: among 892 patients with stable angina and creatinine ≤3 mg/dL, creatinine independently predicted both the severity and the anatomical extent of angiographic coronary disease, and it is best regarded as a marker of the processes that promote atherosclerosis rather than as directly atherogenic [16]. Lower albumin was independently associated with the endpoint in every model, consistent with its established role as a marker of nutritional-metabolic vulnerability and systemic risk in coronary disease [28,29]. The newly examined procedural metrics—access route, fluoroscopy time, radiation dose, stent number, total stent length, bifurcation PCI, and CTO PCI—were not associated with outcome either descriptively (Table 1) or in the augmented multivariable model (Table 5), indicating that these measured procedural variables did not materially attenuate the association between post-PCI hs-CRP and outcome. Calcified-lesion status and IVUS use were not available in the current database. Procedural, lesion, and stent-related factors—including stent type, stent length, number of stents, and lesion complexity—are well-recognized determinants of in-stent restenosis specifically [30,31,32]. Two considerations reconcile this with our null procedural findings. First, our endpoint was a broad composite of death, myocardial infarction, stroke, and repeat revascularization rather than angiographic in-stent restenosis, the outcome those factors most directly predict; pooled restenosis meta-analyses report per-unit associations for stent length (odds ratio 1.026 per millimeter) and number of stents (odds ratio 1.62 per additional stent) [31], derived for angiographic in-stent restenosis in large pooled cohorts, and with only 43 composite events, our study had limited power to detect procedural associations with a broad clinical endpoint. Second, all patients in this contemporary cohort received second-generation drug-eluting stents, so the bare-metal-versus-drug-eluting-stent contrast that drives much of the restenosis literature [30] was not a source of variation here; stent implantation pressure was likewise not recorded.

The prognostic value of post-PCI hs-CRP was also independent of baseline secondary-prevention therapy. Adjustment for pre-procedural statin, aspirin, and P2Y12-inhibitor use left the association essentially unchanged (Supplementary Table S3), and none of these medications were independently associated with outcome. This is relevant because statins lower hs-CRP as well as LDL cholesterol: in the JUPITER trial, rosuvastatin reduced hs-CRP by 37% and lowered the rate of first major cardiovascular events by 44% (hazard ratio 0.56) in apparently healthy individuals selected for an hs-CRP ≥ 2 mg/L despite LDL cholesterol below treatment thresholds [33]. The persistence of an hs-CRP–outcome association despite background statin use is therefore consistent with the concept of residual inflammatory risk—a heightened post-procedural inflammatory response that is not fully abrogated by conventional secondary prevention and that may identify candidates for more intensive or specifically anti-inflammatory strategies.

Taken together, these observations point to a simple, low-cost opportunity to refine risk assessment after elective PCI. Both post-PCI hs-CRP and post-PCI creatinine are inexpensive, universally available laboratory measurements that are already obtained as part of routine post-procedural care, requiring no additional testing, cost, or patient burden: a single blood sample drawn 18 to 24 h after PCI yields two complementary prognostic signals. Because the two markers identified largely non-overlapping high-risk groups, their combined use—together with baseline vulnerability (leukocyte count, albumin, and renal function) and clinical judgment—may stratify risk more effectively than any single variable, and the magnitude of the hs-CRP rise from baseline provides a directly interpretable index of the individual patient’s inflammatory response to the procedure. Two implications follow. First, patients who mount a heightened inflammatory response after an apparently uncomplicated elective procedure and those with a concurrent post-procedural creatinine rise may warrant closer surveillance and more determined secondary prevention rather than routine discharge. Second, in an era when anti-inflammatory therapies—canakinumab in CANTOS and colchicine in LoDoCo2 and COLCOT—have demonstrated cardiovascular benefit, an exaggerated and persistent post-PCI inflammatory response is an attractive, testable criterion for selecting patients who might derive particular benefit from targeted anti-inflammatory treatment, while a rising post-procedural creatinine simultaneously flags those in whom attention to renal protection is most pressing [2,3,24]. These findings should nonetheless be interpreted with appropriate restraint: discrimination for the composite endpoint was modest (AUC approximately 0.62–0.67), so post-PCI hs-CRP and creatinine are best regarded as inexpensive risk-refining adjuncts rather than standalone decision tools, and the associations reported here are hypothesis-generating and require confirmation in prospective, externally validated cohorts before they can guide management.

### Strengths and Limitations

This study has several limitations. First, it was a single-center prospective observational study with a modest number of composite endpoint events (43), which limits generalizability and precludes causal inference; the forced-entry models, while more robust to overfitting than stepwise selection, still analyze 43 events across five to six covariates, at the lower end of conventional events-per-variable recommendations (heuristic shrinkage 0.71–0.75), so point estimates should be interpreted with appropriate caution. Second, the follow-up period was relatively short (median 10 months), so the associations reported here reflect early-to-intermediate outcomes; longer follow-up is needed to establish durability, and the later portions of the Kaplan–Meier curves are based on small numbers at risk and should be interpreted cautiously. Third, regarding the myocardial-infarction component, the infarctions were diagnosed clinically and confirmed at repeat angiography; because the number of acute coronary syndrome events was small (*n* = 16), we did not subclassify them into STEMI and NSTEMI or perform a subgroup analysis by infarction type. Patients with suspected ACS presentation but no angiographically significant culprit lesions were conservatively excluded. Planned/staged PCI procedures were not counted as endpoint events (each patient was included once), consistent with standard practice, since a pre-planned staged procedure represents scheduled care rather than a clinical failure. Fourth, post-PCI creatinine elevation is described as a post-procedural renal-function signal rather than contrast-induced nephropathy because the 18-to-24 h measurement window is earlier than the 48-to-72 h window conventionally used to diagnose that condition. Fifth, the exploratory ROC analyses did not account for censoring or variable follow-up and should be read as secondary descriptive analyses; bootstrap resampling indicated reasonable internal stability, but this is internal, same-sample validation and does not substitute for external validation in an independent cohort. Sixth, patients on chronic dialysis (*n* = 3) were excluded so that creatinine could be interpreted as a graded marker of native glomerular filtration; consequently, the post-PCI renal-function findings do not extend to patients with end-stage renal disease. Finally, this dataset cannot determine whether hs-CRP is a causal mediator, a risk marker, or both, and calcified-lesion status and IVUS use were not available for adjustment. Because this was a single prospective cohort rather than a matched comparison, the two groups differed at baseline as expected; these differences represent risk associations rather than allocation imbalance and were addressed by multivariable adjustment rather than by matching, although residual confounding cannot be excluded. Because patient-level discharge medication use and adherence during follow-up were not captured, we could not validly compare post-PCI pharmacotherapy between outcome groups; the baseline-medication sensitivity analysis (Supplementary Table S3) concerns pre-procedural therapy only and is not a substitute for such a comparison. Finally, all stents were second-generation drug-eluting stents, so these findings do not address stent-type effects, and stent implantation pressure was not captured.

## 5. Conclusions

In this single-center prospective cohort of patients with stable coronary artery disease undergoing elective PCI, higher post-PCI hs-CRP was associated with adverse outcomes independently of baseline hs-CRP and of angiographic and procedural characteristics, indicating that it provided prognostic information beyond baseline inflammatory status rather than merely reflecting it. Post-PCI creatinine was a complementary, largely independent risk signal. Because discrimination was modest and the number of events and the follow-up period were limited, these inexpensive, routinely available markers should be regarded as potential risk-refining adjuncts rather than standalone decision tools. The findings are hypothesis-generating and require confirmation in larger, prospective, externally validated cohorts with longer follow-up.

## Figures and Tables

**Table 1:** Baseline and procedural characteristics according to composite endpoint status.

Variable	Composite Endpoint Absent (*n* = 453)	Composite Endpoint Present (*n* = 43)	Total (*n* = 496)	*p* Value
Age (years)	63.5 ± 9.0	62.7 ± 10.6	63.4 ± 9.2	0.636
Female (*n*, %)	135 (29.8%)	12 (27.9%)	147 (29.6%)	0.932
Diabetes mellitus (*n*, %)	211 (46.6%)	19 (44.2%)	230 (46.4%)	0.888
Hypertension (*n*, %)	358 (79.0%)	36 (83.7%)	394 (79.4%)	0.596
**Hyperlipidemia (*n*, %)**	263 (58.1%)	34 (79.1%)	297 (59.9%)	**0.012**
Prior CAD (*n*, %)	163 (36.0%)	19 (44.2%)	182 (36.7%)	0.368
Prior stroke (*n*, %)	18 (4.0%)	3 (7.0%)	21 (4.2%)	0.414
Smoking (*n*, %)	176 (38.9%)	12 (27.9%)	188 (37.9%)	0.212
Baseline statin (*n*, %)	284 (62.7%)	32 (74.4%)	316 (63.7%)	0.173
Baseline aspirin (*n*, %)	285 (62.9%)	33 (76.7%)	318 (64.1%)	0.101
Baseline P2Y12 inhibitor (*n*, %)	194 (42.8%)	25 (58.1%)	219 (44.2%)	0.076
Ejection fraction (%)	56.9 ± 7.9	54.9 ± 9.2	56.8 ± 8.0	0.202
Pre-PCI hemoglobin (g/dL)	13.4 ± 1.6	13.0 ± 1.7	13.3 ± 1.6	0.233
**Pre-PCI WBC (×10^9^/L)**	7.76 ± 2.02	8.78 ± 2.74	7.85 ± 2.11	**0.022**
**Pre-PCI platelet (×10^9^/L)**	241 (193–278)	265 (219–309)	242 (193–282)	**0.040**
**Pre-PCI creatinine (mg/dL)**	0.89 (0.75–1.03)	0.94 (0.85–1.17)	0.89 (0.76–1.04)	**0.017**
**Albumin (g/L)**	41.5 ± 3.0	40.3 ± 4.0	41.4 ± 3.1	**0.050**
Total cholesterol (mg/dL)	166 (139–201)	167 (148–190)	166 (140–200)	0.700
LDL cholesterol (mg/dL)	101 (78–132)	98 (87–126)	101 (78–130)	0.544
HDL cholesterol (mg/dL)	40.6 ± 10.2	40.2 ± 12.2	40.6 ± 10.4	0.824
Pre-PCI hs-CRP (mg/L)	2.5 (1.1–5.4)	3.1 (1.4–7.5)	2.5 (1.1–5.5)	0.174
Access site–femoral (*n*, %)	93 (20.5%)	11 (25.6%)	104 (21.0%)	0.561
Fluoroscopy time (sec)	634 (444–830)	597 (464–728)	626 (446–830)	0.529
Radiation dose (Gy)	0.73 (0.46–1.06)	0.73 (0.50–0.93)	0.73 (0.47–1.04)	0.938
Fluoroscopy frame count	748 (537–969)	766 (525–926)	754 (536–965)	0.843
LAD PCI (*n*, %)	224 (49.4%)	15 (34.9%)	239 (48.2%)	0.096
CX PCI (*n*, %)	122 (26.9%)	16 (37.2%)	138 (27.8%)	0.208
RCA PCI (*n*, %)	136 (30.0%)	13 (30.2%)	149 (30.0%)	1.000
Saphenous vein graft PCI (*n*, %)	1 (0.2%)	0 (0.0%)	1 (0.2%)	1.000
Bifurcation PCI (*n*, %)	18 (4.0%)	2 (4.7%)	20 (4.0%)	0.689
CTO PCI (*n*, %)	27 (6.0%)	1 (2.3%)	28 (5.6%)	0.497
Predilatation (*n*, %)	296 (65.3%)	24 (55.8%)	320 (64.5%)	0.280
Number of stents	1 (1–2)	1 (1–2)	1 (1–2)	0.833
Total stent length (mm)	29 (21–44)	30 (22–38)	29 (21–43)	0.977
Maximum stent diameter (mm)	3.03 ± 0.43	3.02 ± 0.45	3.03 ± 0.43	0.947
Postdilatation (*n*, %)	266 (58.7%)	23 (53.5%)	289 (58.3%)	0.615
Oversize (*n*, %)	88 (19.4%)	5 (11.6%)	93 (18.8%)	0.295
Post-PCI hemoglobin (g/dL)	12.8 ± 1.6	12.7 ± 1.7	12.8 ± 1.6	0.653
Post-PCI WBC (×10^9^/L)	8.49 ± 1.95	8.98 ± 1.98	8.53 ± 1.95	0.126
**Post-PCI platelet (×10^9^/L)**	227 (186–263)	255 (207–294)	230 (187–267)	**0.008**
**Post-PCI creatinine (mg/dL)**	0.85 (0.73–1.01)	0.92 (0.83–1.10)	0.86 (0.74–1.01)	**0.010**
**Post-PCI hs-CRP (mg/L)**	2.5 (1.2–5.3)	4.3 (2.1–9.8)	2.6 (1.3–5.7)	**0.001**
**Δhs-CRP (mg/L)**	0.0 (−0.3–0.5)	0.7 (−0.1–2.4)	0.0 (−0.3–0.6)	**0.002**
**Post-PCI troponin (ng/L)**	25.0 (14.0–44.0)	31.0 (21.1–59.0)	25.6 (14.2–47.0)	**0.026**

Continuous variables are presented as mean ± SD (compared with Welch’s *t*-test) or median (interquartile range) (compared with the Mann–Whitney U test), as appropriate; categorical variables are presented as *n* (%) (compared with the chi-square test with Yates continuity correction, or Fisher’s exact test when an expected cell count was <5). Bold indicates *p* < 0.05. Troponin is high-sensitivity cardiac troponin (institutional assay, ng/L). CAD, coronary artery disease; CTO, chronic total occlusion; CX, circumflex; HDL, high-density lipoprotein; hs-CRP, high-sensitivity C-reactive protein; LAD, left anterior descending; LDL, low-density lipoprotein; PCI, percutaneous coronary intervention; RCA, right coronary artery; WBC, white blood cell count. Statin, aspirin, and P2Y12 inhibitor denote baseline (pre-procedural) medication use.

**Table 2:** Patient characteristics according to post-PCI hs-CRP elevation (cut-off 1.90 mg/L).

Variable	Post-PCI hs-CRP < 1.90 mg/L (*n* = 188)	Post-PCI hs-CRP ≥ 1.90 mg/L (*n* = 308)	Total (*n* = 496)	*p* Value
Age (years)	63.8 ± 9.0	63.2 ± 9.3	63.4 ± 9.2	0.453
**Female (*n*, %)**	45 (23.9%)	102 (33.1%)	147 (29.6%)	**0.038**
Diabetes mellitus (*n*, %)	84 (44.7%)	146 (47.4%)	230 (46.4%)	0.619
Hypertension (*n*, %)	147 (78.2%)	247 (80.2%)	394 (79.4%)	0.674
Hyperlipidemia (*n*, %)	111 (59.0%)	186 (60.4%)	297 (59.9%)	0.840
Prior CAD (*n*, %)	68 (36.2%)	114 (37.0%)	182 (36.7%)	0.926
Prior stroke (*n*, %)	9 (4.8%)	12 (3.9%)	21 (4.2%)	0.804
Smoking (*n*, %)	68 (36.2%)	120 (39.0%)	188 (37.9%)	0.599
Baseline statin (*n*, %)	124 (66.0%)	192 (62.3%)	316 (63.7%)	0.473
Baseline aspirin (*n*, %)	115 (61.2%)	203 (65.9%)	318 (64.1%)	0.332
Baseline P2Y12 inhibitor (*n*, %)	84 (44.7%)	135 (43.8%)	219 (44.2%)	0.927
Ejection fraction (%)	56.9 ± 8.4	56.7 ± 7.8	56.8 ± 8.0	0.756
Pre-PCI hemoglobin (g/dL)	13.5 ± 1.6	13.2 ± 1.6	13.3 ± 1.6	0.092
**Pre-PCI WBC (×10^9^/L)**	7.34 ± 1.66	8.16 ± 2.29	7.85 ± 2.11	**<0.001**
**Pre-PCI platelet (×10^9^/L)**	230 (188–265)	253 (202–290)	242 (193–282)	**<0.001**
Pre-PCI creatinine (mg/dL)	0.90 (0.76–1.07)	0.89 (0.77–1.03)	0.89 (0.76–1.04)	0.894
**Albumin (g/L)**	41.9 ± 3.0	41.1 ± 3.1	41.4 ± 3.1	**0.006**
Total cholesterol (mg/dL)	163 (137–190)	167 (142–205)	166 (140–200)	0.073
**LDL cholesterol (mg/dL)**	96 (76–125)	102 (82–138)	101 (78–130)	**0.022**
**HDL cholesterol (mg/dL)**	42.6 ± 11.3	39.4 ± 9.5	40.6 ± 10.4	**0.001**
**Pre-PCI hs-CRP (mg/L)**	1.0 (0.6–1.4)	4.2 (2.7–8.4)	2.5 (1.1–5.5)	**<0.001**
Access site–femoral (*n*, %)	36 (19.1%)	68 (22.1%)	104 (21.0%)	0.507
Fluoroscopy time (sec)	623 (407–804)	626 (463–869)	626 (446–830)	0.199
Radiation dose (Gy)	0.73 (0.43–0.97)	0.73 (0.48–1.07)	0.73 (0.47–1.04)	0.241
Fluoroscopy frame count	730 (528–942)	766 (544–969)	754 (536–965)	0.283
LAD PCI (*n*, %)	89 (47.3%)	150 (48.7%)	239 (48.2%)	0.840
CX PCI (*n*, %)	57 (30.3%)	81 (26.3%)	138 (27.8%)	0.386
RCA PCI (*n*, %)	52 (27.7%)	97 (31.5%)	149 (30.0%)	0.422
Saphenous vein graft PCI (*n*, %)	0 (0.0%)	1 (0.3%)	1 (0.2%)	1.000
Bifurcation PCI (*n*, %)	6 (3.2%)	14 (4.5%)	20 (4.0%)	0.611
CTO PCI (*n*, %)	9 (4.8%)	19 (6.2%)	28 (5.6%)	0.655
Predilatation (*n*, %)	121 (64.4%)	199 (64.6%)	320 (64.5%)	1.000
Number of stents	1 (1–2)	1 (1–2)	1 (1–2)	0.605
Total stent length (mm)	26 (21–38)	31 (21–44)	29 (21–43)	0.458
Maximum stent diameter (mm)	3.03 ± 0.43	3.03 ± 0.43	3.03 ± 0.43	0.940
Postdilatation (*n*, %)	115 (61.2%)	174 (56.5%)	289 (58.3%)	0.352
Oversize (*n*, %)	30 (16.0%)	63 (20.5%)	93 (18.8%)	0.260
Post-PCI hemoglobin (g/dL)	12.9 ± 1.5	12.7 ± 1.6	12.8 ± 1.6	0.439
**Post-PCI WBC (×10^9^/L)**	8.24 ± 1.81	8.71 ± 2.02	8.53 ± 1.95	**0.008**
**Post-PCI platelet (×10^9^/L)**	208 (174–247)	241 (200–280)	230 (187–267)	**<0.001**
Post-PCI creatinine (mg/dL)	0.85 (0.74–1.01)	0.86 (0.74–1.01)	0.86 (0.74–1.01)	0.653
**Post-PCI hs-CRP (mg/L)**	1.0 (0.7–1.4)	4.7 (2.8–9.1)	2.6 (1.3–5.7)	**<0.001**
**Δhs-CRP (mg/L)**	0.0 (−0.2–0.2)	0.2 (−0.5–1.2)	0.0 (−0.3–0.6)	**0.005**
Post-PCI troponin (ng/L)	23.6 (13.1–48.1)	26.1 (15.0–47.0)	25.6 (14.2–47.0)	0.180

Continuous variables are presented as mean ± SD (Welch’s *t*-test) or median (interquartile range) (Mann–Whitney U test); categorical variables are *n* (%) (chi-square test with Yates continuity correction, or Fisher’s exact test for expected counts <5). Bold indicates *p* < 0.05. The 1.90 mg/L threshold is the Youden-optimal cut-off for post-PCI hs-CRP derived from ROC analysis in this cohort. Abbreviations same as in Table 1.

**Table 3:** Pre-PCI multivariable Cox model for the composite endpoint.

Variable	Hazard Ratio (95% CI)	*p* Value
Age (per year)	1.003 (0.968–1.038)	0.879
Female sex	1.078 (0.534–2.173)	0.834
**Pre-PCI creatinine (per 0.1 mg/dL)**	1.152 (1.059–1.253)	**0.001**
**Albumin (per g/L)**	0.912 (0.836–0.996)	**0.040**
**Pre-PCI WBC (per ×10^9^/L)**	1.152 (1.018–1.304)	**0.025**

Forced-entry multivariable Cox proportional-hazards model (*n* = 496, 43 events). Creatinine is reported per 0.1 mg/dL because its interquartile range in this cohort is far narrower than 1.0 mg/dL; after exclusion of the three chronic-dialysis patients, it was neither markedly influential nor non-linear and satisfied the proportional-hazards assumption (Supplementary Table S1). CI, confidence interval. Bold indicates *p* < 0.05.

**Table 4:** Post-PCI multivariable Cox model for the composite endpoint (adjusted for baseline hs-CRP).

Variable	Hazard Ratio (95% CI)	*p* Value
Age (per year)	1.000 (0.967–1.034)	0.995
Female sex	0.882 (0.439–1.772)	0.725
**Post-PCI creatinine (per 0.1 mg/dL)**	1.156 (1.059–1.261)	**0.001**
**Albumin (per g/L)**	0.882 (0.806–0.965)	**0.006**
Pre-PCI hs-CRP (per doubling)	0.763 (0.562–1.036)	0.083
**Post-PCI hs-CRP (per doubling)**	1.432 (1.047–1.960)	**0.025**

Forced-entry multivariable Cox proportional-hazards model (*n* = 496, 43 events). hs-CRP is strongly right-skewed and was analyzed on the log scale (hazard ratio per doubling, log_2_), its conventional analytic form for this biomarker; [19,20,21,22] on the untransformed scale, it also violated the proportional-hazards assumption, which the log scale resolves. Creatinine is reported per 0.1 mg/dL because its interquartile range in this cohort is far narrower than 1.0 mg/dL; after exclusion of the three chronic-dialysis patients, it was neither markedly influential nor non-linear and satisfied the proportional-hazards assumption (Supplementary Table S1). CI, confidence interval. Bold indicates *p* < 0.05.

**Table 5:** Post-PCI model augmented with angiographic and procedural characteristics.

Variable	Hazard Ratio (95% CI)	*p* Value
Age (per year)	1.005 (0.970–1.040)	0.794
Female sex	0.818 (0.402–1.666)	0.580
**Post-PCI creatinine (per 0.1 mg/dL)**	1.171 (1.066–1.286)	**0.001**
**Albumin (per g/L)**	0.879 (0.804–0.962)	**0.005**
Pre-PCI hs-CRP (per doubling)	0.740 (0.544–1.008)	0.056
**Post-PCI hs-CRP (per doubling)**	1.540 (1.118–2.122)	**0.008**
Number of stents (per stent)	0.783 (0.322–1.902)	0.589
Total stent length (per mm)	0.992 (0.958–1.027)	0.635
Bifurcation PCI	1.057 (0.234–4.771)	0.942
CTO PCI	0.267 (0.035–2.050)	0.204

Forced-entry multivariable Cox proportional-hazards model (*n* = 496, 43 events). Procedural covariates (number of stents, total stent length, bifurcation PCI, CTO PCI) added to the Table 4 model. hs-CRP is strongly right-skewed and was analyzed on the log scale (hazard ratio per doubling, log_2_), its conventional analytic form for this biomarker; [19,20,21,22] on the untransformed scale, it also violated the proportional-hazards assumption, which the log scale resolves. Creatinine is reported per 0.1 mg/dL because its interquartile range in this cohort is far narrower than 1.0 mg/dL; after exclusion of the three chronic-dialysis patients, it was neither markedly influential nor non-linear and satisfied the proportional-hazards assumption (Supplementary Table S1). CI, confidence interval. Bold indicates *p* < 0.05.

## Data Availability

The de-identified data supporting the findings of this study are available from the corresponding author upon reasonable request. Data containing patient-identifying information cannot be shared owing to privacy and ethical restrictions.

## Supplementary Materials

The following supporting information can be downloaded at: https://www.mdpi.com/article/10.3390/jcm15176557/s1, Table S1: Proportional-hazards assumption (Schoenfeld residuals, Grambsch-Therneau); Table S2: ROC discrimination with bootstrap internal validation; Table S3: Post-PCI model additionally adjusted for baseline medication use.
