# Supplementary material for: Inflammatory Response After Elective PCI and Subsequent Outcomes in Stable Coronary Artery Disease
Source: J Clin Med. 2026 Aug 25;15(17):6557. doi: 10.3390/jcm15176557 (PMC13566776; doi:10.3390/jcm15176557)
Supplement: Supplementary file 1 [file jcm-15-06557-s001.zip › jcm-4485913-supplementary.pdf]

Supplementary Materials

Supplementary Table S1. Proportional-Hazards Assumption (Schoenfeld Residuals, Grambsch-Therneau)

Pre-PCI model

Covariate	ρ (residual–time)	χ^2	P value
Age	-0.270	3.56	0.059
Female sex	+0.240	2.40	0.122
Pre-PCI creatinine	+0.163	1.00	0.318
Albumin	+0.210	2.39	0.122
Pre-PCI WBC	+0.044	0.10	0.751
**Global test**	—	7.60 (5 df)	0.180

Post-PCI model

Covariate	ρ (residual–time)	χ^2	P value
Age	-0.242	2.93	0.087
Female sex	+0.171	1.20	0.273
Post-PCI creatinine	+0.289	3.33	0.068
Albumin	+0.125	0.68	0.408
Pre-PCI hs-CRP (log)	-0.120	0.64	0.422
Post-PCI hs-CRP (log)	-0.005	0.00	0.975
**Global test**	—	8.89 (6 df)	0.180

Scaled Schoenfeld residuals were correlated with the rank of survival time; per-covariate χ^2 (1 df) and a global χ^2 (df = number of covariates) test the null of proportional hazards. In these models hs-CRP is entered on the log scale and creatinine on its natural (mg/dL) scale. No individual covariate and neither global test showed a significant departure from proportional hazards (all $p > 0.05$). Log-linearity of log-hs-CRP and linearity of creatinine were supported by non-significant quadratic terms (pre-PCI creatinine $p = 0.359$, post-PCI creatinine $p = 0.335$, post-PCI log-hs-CRP $p = 0.235$). After exclusion of the 3 chronic-dialysis patients, no observation exceeded the conventional standardized-dfbeta threshold of 1.0 (maximum 0.603) and all association directions were preserved on leave-one-out refitting. Given the events-per-variable (7.2–8.6), the heuristic shrinkage factor was 0.709–0.753 and the bootstrap optimism-corrected concordance index was 0.649 (apparent 0.686), indicating limited overfitting.

Supplementary Table S2. ROC Discrimination with Bootstrap Internal Validation (2000 resamples)

Biomarker	Apparent AUC	Bootstrap 95% CI	Youden cut-off	Sensitivity	Specificity
Pre-PCI WBC	0.606	0.514–0.690	7.83	62.8%	57.2%
Post-PCI hs-CRP	0.651	0.572–0.729	1.90	86.0%	40.2%
Δhs-CRP (post − pre)	0.642	0.531–0.744	0.50	60.5%	74.4%
Pre-PCI hs-CRP	0.563	0.475–0.659	1.90	72.1%	41.9%

Exploratory ROC analysis for the composite endpoint (n = 496). The bootstrap 95% CI (percentile method, 2000 resamples) quantifies internal precision; this is internal, not external, validation. Creatinine was analyzed as a continuous Cox covariate rather than via a threshold and is not shown. Units: WBC $\times 10^9$/L; hs-CRP mg/L.

Supplementary Table S3. Post-PCI Model Additionally Adjusted for Baseline Medication Use (Sensitivity Analysis)

Variable	Hazard ratio (95% CI)	P value
Age (per year)	0.993 (0.958–1.030)	0.712
Female sex	1.134 (0.541–2.374)	0.739
**Post-PCI creatinine (per 0.1 mg/dL)**	1.149 (1.046–1.262)	**0.004**
**Albumin (per g/L)**	0.879 (0.803–0.964)	**0.006**
**Pre-PCI hs-CRP (per doubling)**	0.742 (0.554–0.992)	**0.044**
**Post-PCI hs-CRP (per doubling)**	1.471 (1.088–1.988)	**0.012**
Baseline statin	1.208 (0.524–2.784)	0.658
Baseline aspirin	1.831 (0.827–4.052)	0.136
Baseline P2Y12 inhibitor	1.582 (0.762–3.287)	0.219

Forced-entry Cox model (n = 496, 43 events): the post-PCI model of Table 4 with baseline statin, aspirin, and P2Y12-inhibitor use added. Post-PCI hs-CRP and post-PCI creatinine remained independently associated with the composite endpoint, and none of the three medications was independently associated, indicating the primary associations are not explained by baseline secondary-prevention medication use. hs-CRP hazard ratios are per doubling ($\log_2$); creatinine per 0.1 mg/dL. CI, confidence interval. Bold indicates $p < 0.05$.
